# Isoflurane pre-treatment before cardiopulmonary bypass alleviates neutrophil accumulation in dog lungs

**DOI:** 10.5830/CVJA-2010-055

**Published:** 2011-06

**Authors:** Gui-Zhi Du, Jin Liu, Hong Gao, Xiang-Gang Zeng, Xiang He, Guan-Sheng Wu, Xuan-Yi Hu, Xin-Hua Li

**Affiliations:** Department of Anesthesiology, West China Hospital, Sichuan University, Chengdu 610041, Sichuan, China; Department of Anesthesiology, West China Hospital, Sichuan University, Chengdu 610041, Sichuan, China; Department of Anesthesiology, the Affiliated Hospital of Guiyang Medical College, Guiyang 550004, Guizhou, China; Department of Anesthesiology, the Affiliated Hospital of Guiyang Medical College, Guiyang 550004, Guizhou, China; Department of Anesthesiology, Guizhou Provincial Hospital, Guiyang 550002, Guizhou, China; Department of Cardiac Surgery, the Affiliated Hospital of Guiyang Medical College, Guiyang 550004, Guizhou, China; Department of Cardiac Surgery, the Affiliated Hospital of Guiyang Medical College, Guiyang 550004, Guizhou, China; Division of Public Health, Guiyang Medical College, Guiyang 550004, Guizhou, China

**Keywords:** cardiopulmonary bypass, lung injury, isoflurane, dog

## Abstract

**Objective:**

This study investigated the effect of isoflurane pre-treatment on cardiopulmonary bypass (CPB)-related lung injury.

**Methods:**

Twelve dogs were randomly divided into two groups of six each. In one group, 1.0 minimum alveolar concentration (MAC) of isoflurane was administered for 30 min before CPB, while the control group received no anaesthetic. Both groups then underwent 100 min of mild hypothermic CPB with 60-min aortic cross clamping. Haemodynamic parameters, respiratory mechanics and alveolar arterial oxygen difference (AaDO_2_) were measured during the experiment. One hundred and fifty minutes after CPB, lung tissue samples from the non-dependent and dependent portions of the left and right lungs were harvested for polymorphonulear leukocyte (PMNs) counts.

**Results:**

Following CPB, within the control group, pulmonary vascular resistance (PVR) was significantly increased at 60, 120 and 180 min after declamping, AaDO2 deteriorated at 180 min post-declamping, and dynamic lung compliance (DLC) was reduced dramatically after declamping. Isoflurane pre-treatment before CPB significantly reduced PVR compared to the controls. AaDO_2_ was impaired at 180 min after declamping and DLC was decreased after declamping within the isoflurane group. No differences in AaDO_2_ and DLC were found between the isoflurane and control groups. At 180 min after declamping, the PMN count in both the non-dependent and dependent regions of the isoflurane pre-treated lungs was significantly lower than that of the controls.

**Conclusions:**

Our results suggest that 30-min pre-treatment with 1.0 MAC isoflurane before CPB caused a reduction in PMN accumulation in the dog lungs, inhibition of increases in PVR, and it did not affect AaDO2 in the early post-CPB stage.

## Abstract

The lungs are one of the most susceptible organs to ischaemia/reperfusion (IR) injury and systemic inflammatory response syndrome (SIRS) initiated during cardiopulmonary bypass (CPB).^1^ Laboratory and clinical investigations have demonstrated that accumulation of polymorphonuclear leukocytes (PMNs) in the lungs plays a key role in the pathogenesis of CPB-related lung injury, which leads to impairment of gas exchange, increase in pulmonary vascular resistance (PVR) and decrease in pulmonary compliance.^1^-^5^

Volatile anaesthetics are commonly used during cardiac surgery. Nevertheless the effects of inhaled anaesthetic agents on lung injury remain controversial. Some studies showed that volatile anaesthetics can enhance the sensitivity of pulmonary artery endothelial cells to oxidation-mediated injury,^6^ heighten pulmonary alveolar capillary membrane permeability in rabbits after aortic occlusion-perfusion,^7^ and augment acute inflammatory response and leukocytic infiltration in acid-impaired rat lungs,^8^ indicating that volatile anaesthetics may potentially exacerbate lung injury.

By contrast, in a mouse model of multiple organ dysfunction syndrome, it was shown that halothane and isoflurane attenuated lung inflammation and injury.^9^ Liu and colleagues^10^, ^11^ reported the administration of isoflurane before ischaemia and during reperfusion inhibited IR-induced isolated rabbit lung injury and demonstrated that not only isoflurane but also sevoflurane administered before ischaemia attenuated IR-induced injury in isolated rat lungs. More recently, Reutershan^12^ and Li^13^ documented the protective pre-treatment effects by isoflurane against endotoxin-induced lung injury in mouse and rat models.

In addition, the protective effects against IR injury by volatile anaesthetics have been shown in the myocardium,^14^ brain,^15^ liver^16^ and kidney.^17^ These data suggest that inhaled anaesthetics may not necessarily exacerbate lung injury after CPB. We therefore used an adult mongrel dog model of CPB to verify the hypothesis that pre-treatment of 30-min 1.0 minimum alveolar concentration (MAC) isoflurane before CPB would not aggravate lung injury related to CPB during the early post-CPB stage.

## Methods

All experimental procedures used in this investigation were reviewed and approved by the Animal Care and Experimental Committees of Guiyang Medical College and Huaxi Medical School of Sichuan University.

## Surgical preparation and cardiopulmonary bypass

The studies were performed in 12 adult mongrel dogs weighing 10–18 kg. The animals were anaesthetised with intravenous pentobarbital sodium (25 mg/kg as an initial bolus and then 4 mg/kg/h), paralysed with intravenous vecuronium (0.1 mg/kg as an initial bolus and then 50 μg/kg every 30–40 min during the experiment). They were endotracheally intubated and ventilated on a volume-controlled mode with a tidal volume (TV) of 12 ml/kg, an inspiratory:expiratory ratio (I:E) of 1:2, extrinsic positive end-expiratory pressure (PEEP_e_) of 0 cm H_2_O, and an inspired O_2_ fraction (FiO_2_) of 1.0. Respiratory rate (RR) was adjusted to maintain normocapnia as guided by end-tidal CO_2_ monitoring and intermittent arterial blood gas analyses. Peak inspiratory pressure (PIP), intrinsic positive end-expiratory pressure (PEEP_i_) and mean airway pressure (MPaw) were continually measured by the airway monitor system of the anaesthesia machine (Narkomed GS; Dräger Medical Inc, Telford, PA).

The femoral vein and artery were cannulated to administer drugs and fluids, monitor arterial pressure and collect blood samples. A 7-French Swan–Ganz catheter (CritiCath™ SP107H-14 TD Catheter; Becton Dickinson Critical Care Systems, Singapore) was inserted through the right external jugular vein and placed in the main pulmonary artery to record central venous pressure (CVP), pulmonary artery pressure (PAP), and cardiac output (CO) by thermodilution technique (Spacelabs Monitor model 90369; Spacelabs Medical Inc, Redmond, WA). Nasopharyngeal temperature was continuously recorded.

For CPB, the bypass circuit consisted of a heat exchanger, cardiotomy reservoir, roller pump, bubble oxygenator, and microfilter primed with Ringer lactate solution (30 ml/kg), 6% HAES-steril (HES 200/0.5; 20 ml/kg), 30 ml 5% sodium bicarbonate, and heparin (150 U/kg). Gas flow to the oxygenator was 100% oxygen at 1 l/min. After median sternotomy and heparinisation (300 U/kg, IV), the ascending aorta and the right atrium were cannulated for initiation of bypass, and the left atrium for left atrial pressure (LAP) monitoring. The time between commencement of CPB and cardiac plegia arrest was standardised at 10 min. The aorta was clamped in combination with the pulmonary artery for 60 min to prevent any antegrade flow to the lungs and therefore make the ischaemic degree of the lungs comparable.

Ventilation was terminated during the period from cross clamping to declamping. The heart was arrested via the aortic root with cold (4°C) crystalloid St Thomas’ solution (20 ml/kg) at a pressure of 70 mmHg immediately and 30 min after cross clamping. During total CPB, the pump flow was maintained between 80 and 100 ml/kg/min at a mean perfusion pressure of 40–80 mmHg and each animal was cooled to a nasopharyngeal temperature of 28–30°C. Alpha-stat pH management was observed during CPB. Twenty minutes before declamping, rewarming was initiated until the nasopharyngeal temperature of each animal was rewarmed to 38°C before termination of CPB. The heart was defibrillated with 20 J if necessary after cross clamp removal.

At the end of total CPB, the lungs were inflated manually for 15 s to 40 cm H_2_O, according to a technique by Magnusson, *et al*.^18^ Thirty minutes after declamping, all animals were weaned from CPB using dopamine, starting at 3 μg/kg/min and up to 8 μg/kg/min. Following cessation of CPB, the blood in the oxygenator was transfused back into the circulation and all the dogs without heart decannulation and chest closure were closely observed for 150 min. Then lung tissue samples were taken after administration of an overdose of pentobarbital sodium (50 mg/kg, IV). Twelve lung tissue samples of approximately 1 cm × 1 cm × 1 cm were harvested from the non-dependent and dependent portions of the upper, middle and lower lobes of each animal’s left and right lungs, with the excised lungs were inflated to a constant pressure of 20 cm H_2_O. Harvested lung tissue samples were used for PMN counts.

## Experimental protocol

Twelve adult mongrel dogs were randomly divided into two groups (*n* = 6 each). Measurable variables recorded after 15-min stabilisation following heart cannulation served as baseline. During the 40-min period from recording the baseline to initiating CPB, the animals in the control group received no treatment, while the other six dogs received 30 min 1.0 MAC isoflurane (Abbott Laboratories, North Chicago, IL; 1.39% for 1.0 MAC in dogs;^19^ Spacelabs). The pre-treatment time of the clinically frequent applied concentration (1.0 MAC) of isoflurane was based on previous *in vivo* studies concerning myocardial protective preconditioning in dogs.^20^

Dynamic lung compliance (DLC), measured at baseline and 5, 31, 60, 120 and 180 min after declamping, was calculated using the following formula:

$DLC\;(ml/cm\;H₂O)\;=\;\frac{TV\;(ml)}{PIP\;\left(cm\;H₂O\right)-PEEP\;(cm\;H₂O)\;\;}$

Pulmonary vascular resistance, measured at baseline and 31, 60, 120 and 180 min after declamping, was calculated using the following formula:

PVR (dynes/s/cm^5^) = [mean PAP (mm Hg) – mean LAP (mm Hg)] × 79.92/CO (l/min)

Arterial blood samples, taken at baseline and 5, 31 and 180 min after declamping, were examined in a blood gas analyser (i-Stat; Abbott Laboratories Inc., East Windsor, NJ). Alveolar arterial oxygen difference (AaDO_2_) was calculated using the following formula:

$AaD0₂\;(mmHG)=\frac{Pi0₂\;-PaC0₂\;}{R-Pa0₂}$

PiO_2_ = (P_B_ – P_H2O_) × FiO_2_

Where PiO_2_ is inspired oxygen pressure, R is respiratory quotient with an assumed value of 0.8, P_B_ is barometric pressure with an assumed value of 760, P_H2O_ is the pressure of the water vapour at body temperature with an assumed value of 47, and PaO_2_ is arterial oxygen tension.

The excised lung tissue samples were immediately fixed in 10% formol, then dehydrated, embedded in paraffin, cut into 4-μm slices along a coronal plane from apex to base and stained with haematoxylin and eosin. The slices were coded and examined in a blinded manner by a single examiner. Interstitial and intra-alveolar PMNs were identified and counted in 10 different fields, excluding airways and pulmonary vessels, under 400 × magnification (Model CH30RF200, Olympus, Tokyo, Japan). The data were expressed as number of PMNs per high-power lung field.^21^

## Statistical analysis

Data in the text, tables and figures are expressed as mean ± SEM. Lung tissue PMN counts were evaluated by independent samples *t*-test. Changes in haemodynamic parameters, respiratory mechanics, PVR and AaDO_2_ over time between groups were determined by repeated-measures analysis of variance (ANOVA). Intra-group data at any given time point for a given continuous variable were compared with a respective baseline using the Dunnett *post hoc* test when univariate ANOVA for repeated measures was significant. Additionally, independent samples *t*-tests were used to compare the parameters at each time point between the two groups. Statistical analysis was performed with SPSS software package, version 11.5 (SPSS Inc., Chicago, IL). Significance was assumed at *p* < 0.05.

## Results

Changes in haemodynamic variables are shown in Table 1. MAP, HR, CVP, MPAP, mean LAP and CO values did not change significantly between the groups during the experiment. HR and CO measured at 5 min after declamping were not analysed because of comparatively lower cardiac function at the time. CVP and mean LAP in both groups were within the physiological range at the end of the experiment.

**Table 1.** Haemodynamic Variables

		*Time after aortic declamping (min)*				
*Parameter*	*Baseline*	*5*	*31*	*60*	*120*	*180*
Mean arterial pressure (mmHg)						
Control	82.5 ± 5.5	66.2 ± 6.6	75.5 ± 5.8	60.7 ± 4.9*	59.2 ± 2.8*	57.0 ± 3.8**
Isoflurane	85.8 ± 6.9	57.0 ± 4.5**	61.0 ± 3.1**	64.3 ± 7.6	63.8 ± 6.8	60.2 ± 6.1*
Heart rate (beats/min)						
Control	161.0 ± 9.8		135.7 ± 11.3	119.8 ± 10.2**	98.5 ± 5.9***	91.5 ± 5.1***
Isoflurane	168.5 ± 8.1		140.7 ± 13.0	137.3 ± 9.5	118.5 ± 5.7**	101.3 ± 5.5***
Central venous pressure (mmHg)						
Control	5.7 ± 0.7	4.5 ± 0.9	6.7 ± 1.0	6.6 ± 0.7	7.5 ± 1.3	6.7 ± 0.6
Isoflurane	7.1 ± 0.6	6.6 ± 0.7	6.5 ± 0.7	7.0 ± 0.5	6.7 ± 0.4	7.9 ± 0.4
Mean pulmonary artery pressure (mmHg)						
Control	10.2 ± 1.2	12.2 ± 1.2	15.7 ± 2.6	15.8 ± 2.0	15.0 ± 1.6	14.7 ± 0.6
Isoflurane	10.7 ± 1.7	7.8 ± 1.9	13.8 ± 2.0	13.3 ± 1.1	14.7 ± 0.9	12.8 ± 1.5
Mean left atrial pressure (mmHg)						
Control	5.3 ± 1.2	4.7 ± 0.8	6.5 ± 1.2	5.7 ± 1.4	7.7 ± 1.9	6.3 ± 1.5
Isoflurane	6.2 ± 1.9	4.2 ± 1.5	6.0 ± 1.2	5.3 ± 0.5	8.0 ± 1.2	7.2 ± 1.3
Cardiac output (l/min)						
Control	3.7 ± 0.4		4.0 ± 0.5	3.8 ± 0.5	2.6 ± 0.5	3.0 ± 0.6
Isoflurane	3.3 ± 0.5		3.6 ± 0.6	3.4 ± 0.5	3.0 ± 0.5	2.6 ± 0.4

Data shown are mean ± SEM. *p < 0.05, **p < 0.01 and ***p < 0.001 compared with baseline.

At baseline, PIP, MPaw, PEEP_i_ and DLC values in the group treated with isoflurane were comparable to those in the control group. Following declamping, PIP and MPaw were significantly increased, and DLC decreased in both the isoflurane and control groups. Isoflurane pre-treatment did not affect PIP, MPaw, PEEP_i_ and DLC significantly, compared to the controls (Table 2).

**Table 2.** Respiratory Mechanics

		*Time after aortic declamping (min)*				
*Parameter*	*Baseline*	*5*	*31*	*60*	*120*	*180*
Peak inspiratory pressure (cm H_2_O)						
Control	10.8 ± 1.1	16.3 ± 1.5	17.5 ± 1.8*	17.8 ± 1.9*	19.2 ± 2.0**	19.3 ± 1.9**
Isoflurane	9.5 ± 0.4	13.8 ± 1.1	15.2 ± 1.5**	16.2 ± 1.4**	17.5 ± 1.2***	18.3 ± 1.1***
Mean airway pressure (cm H_2_O)						
Control	4.7 ± 0.3	7.0 ± 0.5	7.5 ± 0.7*	7.5 ± 0.7*	7.8 ± 0.7**	7.7 ± 0.7**
Isoflurane	4.0 ± 0.3	5.3 ± 0.2*^#^	5.8 ± 0.3**	6.2 ± 0.4***	6.7 ± 0.2***	6.7 ± 0.4***
Intrinsic positive end expiratory pressure (cm H_2_O)						
Control	1.3 ± 0.2	1.5 ± 0.2	1.5 ± 0.2	1.5 ± 0.2	1.8 ± 0.2	1.8 ± 0.2
Isoflurane	1.3 ± 0.2	1.0 ± 0.3	1.2 ± 0.2	1.3 ± 0.2	1.3 ± 0.2	1.7 ± 0.5
Dynamic lung compliance (ml/cm H_2_O)						
Control	27.7 ± 4.0	16.8 ± 1.7*	14.6 ± 2.8**	14.3 ± 2.1**	13.3 ± 1.9**	12.4 ± 2.1**
Isoflurane	25.5 ± 1.6	16.4 ± 2.1**	14.1 ± 1.8***	13.6 ± 1.5***	13.6 ± 1.5***	11.2 ± 1.1***

Data shown are mean ± SEM. *p < 0.05, **p < 0.01 and ***p < 0.001 compared with baseline. ^#^*p* < 0.05 compared with control at a given time point.

**Fig. 1.**
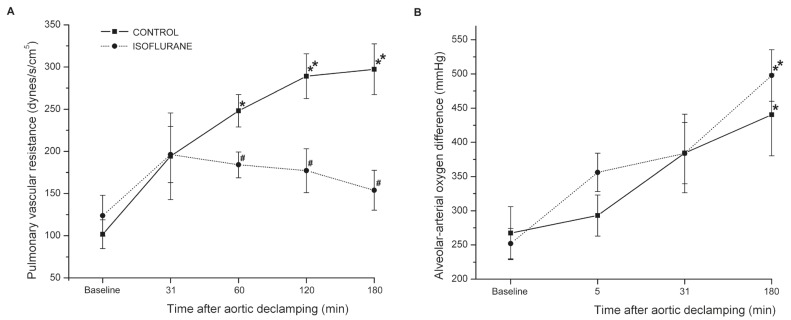
Changes in pulmonary vascular resistance (PVR) at baseline and 31, 60, 120 and 180 min after aortic declamping (A), and alveolar arterial oxygen difference (AaDO_2_) at baseline and 5, 31 and 180 min after aortic declamping (B), of the control and isoflurane groups. Data shown are mean ± SEM. *p < 0.05 vs baseline and **p < 0.01 vs baseline; ^#^*p* < 0.05 compared with control at a given time point.

Fig. 1A reflects the pattern of PVR changes in each group during the study. At baseline, PVR in the isoflurane group was comparable to that in the control. Following CPB, in the control group, PVR was significantly increased at 60 (*p* < 0.05 vs baseline), 120 (*p* < 0.01 vs baseline) and 180 min (*p* < 0.01 vs baseline) after declamping. No differences in PVR were found within the isoflurane group (*p* > 0.05 vs baseline). There were significant differences in PVR between the isoflurane and control groups (*p* < 0.05). At 60, 120 and 180 min after declamping, PVR values were lower in the isoflurane group compared to those in the control group (*p* < 0.05).

Fig. 1B shows the changes in AaDO_2_ in the isoflurane and control groups over the experimental time. At baseline, AaDO_2_ did not differ between the isoflurane and control groups (*p* = 0.429). Within both the isoflurane and the control group, AaDO_2_ at 180 min after declamping was markedly higher than that at baseline (*p* < 0.01 and *p* < 0.05, respectively). Pre-treatment of isoflurane did not affect AaDO_2_ significantly over the experiment, compared to the control group (*p* > 0.05).

**Fig. 2.**
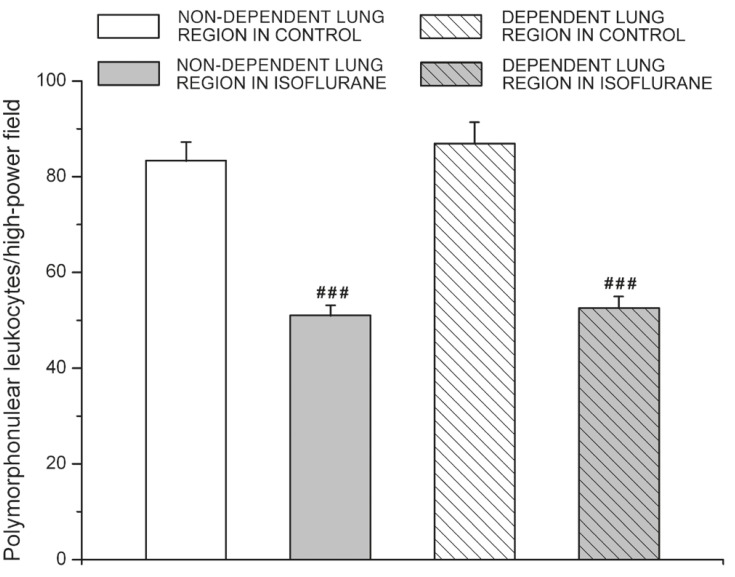
Lung tissue polymorphonuclear leukocyte (PMN) counts (expressed as PMNs/field) in 10 different fields, excluding airways and pulmonary vessels under 400 × magnification in non-dependent and dependent lung regions of the control and isoflurane groups. Data shown are mean ± SEM. ^###^*p* < 0.001 vs control.

**Fig. 3.**
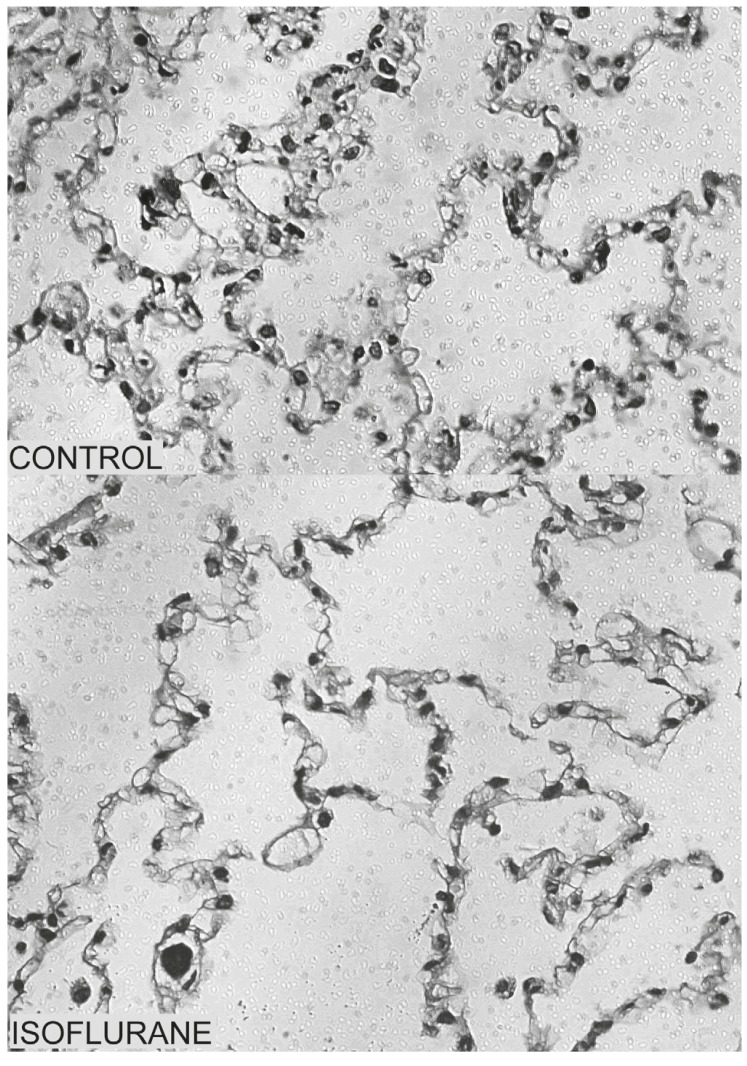
Microscopic findings of polymorphonuclear leukocyte (PMN) filtration in the dependent portion of the lower lobe of the left lung three hours after aortic declamping in the control and isoflurane groups (haematoxylin-eosin; magnification × 400).

PMNs infiltrations in both the non-dependent and dependent lung regions were significantly lower in the isoflurane group compared to those in the controls (both *p* < 0.001). No differences in PMN counts were detected between the non-dependent and dependent lung regions or between left and right lungs in each group (*p* > 0.05) (Figs 2 and 3).

## Discussion

Mechanisms of lung injury and respiratory complications following CPB are multi-factorial, including aspects due to CPB.^22^ During CPB, blood contact with the artificial surface of the bypass circuit, coupled with IR injury contribute to the initiation of SIRS which is partially attributed to activation of PMNs with up-regulation of adhesion molecules. This process may ultimately result in adhesion of PMNs to the endothelium of the lung vessels, and endothelial damage due to protease release.^1^ Researchers in multi-disciplinary fields have been attempting to find better lung protective and therapeutic strategies against CPB-associated lung injury. Few currently developed strategies, however, have been demonstrated to be clinically beneficial.^22^

Recently, some literature reports that volatile anaesthetics showed protective effects against lung injury.^9^-^13^ In a male mouse model of endotoxin-induced lung injury, isoflurane pre-treatment significantly attenuated PMN recruitment into the injured lung.^12^ The clinical dilemma of lung injury during CPB and this encouraging *in vitro* and *in vivo* evidence gave us insight into conducting the experiment. The primary findings of the current experiment were that isoflurane pre-treatment before CPB alleviated PMN accumulation in the lungs, reduced PVR, and did not affect AaDO_2_ during the early post-CPB stage, compared to the controls.

Reduced static and dynamic compliance is one of the characteristics of pulmonary function in patients after exposure to CPB.^23^,^24^ Postulated causes include atelectasis, pulmonary oedema, inflammation, increased capillary permeability, and pleural effusions.^25^ Dogs pre-treated with isoflurane in our experiment showed significantly reduced DLC values after CPB, as did the control group. However, no difference in DLC was found between the two groups. Consistent with our DLC results, in an oleic acid-induced lung injury model, it was also documented that lung compliance of the injured canine lungs was not affected by inhalation of isoflurane.^26^

In the present study we found 30-min pre-treatment with 1.0 MAC isoflurane before CPB significantly decreased PVR in the early post-CPB stage. The effect of isoflurane on PVR shown in our study may potentially be ascribable to the demonstrated phenomenon that isoflurane attenuates hypoxic pulmonary vasoconstriction (HPV).^27^-^30^ It has been shown that metabolites of the cyclooxygenase pathway,^28^ endogenous vasodilator,^29^ calcium-activated potassium (K_Ca_) channels and voltage-dependent potassium (KV) channels^30^ may be involved in the modulation of isoflurane-induced attenuation of HPV. More recently, it has been reported that TASK-1 channels play a role in HPV and contribute to volatile anaesthetic-induced pulmonary vasodilation.^31^

In our experiment, although it was striking that gas exchange in the isoflurane group, assessed by AaDO_2_ changes, deteriorated steadily following the diminution of PVR after aortic declamping, no difference in AaDO_2_ was found between the isoflurane-treated and control groups. The reason might be that the inhibition of HPV by isoflurane caused only minor effects on gas exchange in our study setting. In an oleic acid-induced lung injury model, it has been demonstrated that low concentrations of isoflurane resulted in an increase in ventilation and perfusion mismatch, as evaluated by multiple inert gas analysis.^26^ However, AaDO_2_ in the injured lungs was not significantly changed by inhalation of the low concentrations of isoflurane.^26^

By contrast, in a porcine model with gas-exchange defect by air pneumoperitoneum, it was shown that sevoflurane but not isoflurane caused significant ventilation and perfusion mismatch using the multiple inert gas elimination technique.^32^ The differences between the experimental model (oleic acid-induced vs air pneumoperitoneum-induced gas-exchange defect) and the controls (sodium pentobarbital-anaesthetised animals with definite lung injury vs propofol-anaesthetised animals with gas-exchange defect) may account for the inconsistence shown in the abovementioned two experiments regarding the effect of isoflurane on gas-exchange disturbance.

It is well known that PMNs play a key role in the development of post-CPB lung injury.^1^-^5^,^22^ Previous studies showed that volatile anaesthetics reduced PMN adhesion in the reperfused coronary system.^33^,^34^ Recently it was demonstrated that isoflurane pre-treatment attenuated PMN accumulation in the lung interstitium and alveolar space in a male mouse model of lipopolysaccharide-induced lung injury.^12^ Our results of PMN counts showed that isoflurane remarkably alleviated PMN filtration into the non-dependent and dependent lung regions three hours after declamping, indicating that isoflurane could be beneficial for inhibition of inflammation in lungs during CPB.

Some limitations of our experiment should be noted. First, pentobarbital showed an inhibitary effect on HPV.^35^ Consequently, the effects of isoflurane on CPB-related lung injury may be altered with the pentobarbital anaesthesia. However, the results should be reliable since a continuous quantitative infusion (25 mg/kg as an initial bolus and then 4 mg/kg/h) of pentobarbital was performed in the current study. In addition, to prevent any antegrade flow to the lungs and therefore make any lung injury in each animal as similar as possible, our animals were subject to pulmonary artery as well as aortic clamping during CPB. This may have aggravated the degree of lung injury, in that exposure to CPB alone is enough to cause pulmonary injury, and cessation of pulmonary arterial flow during CPB contributes significantly to pulmonary dysfunction.^24^

## Conclusion

The present study showed that 30-min pre-treatment with 1.0 MAC isoflurane before CPB alleviated PMN accumulation in canine lungs, inhibited the increase in PVR, and did not significantly affect AaDO_2_ in the early post-CPB stage, suggesting isoflurane could be beneficial in preventing CPB-related lung injury.
